# Anti-Inflammatory and Analgesic Effect of Arachic Acid Ethyl Ester Isolated from Propolis

**DOI:** 10.1155/2020/8797284

**Published:** 2020-05-03

**Authors:** Sélestin Dongmo Sokeng, Emmanuel Talla, Paul Sakava, Michel Archange Fokam Tagne, Celine Henoumont, Laurent Sophie, Joseph Tanyi Mbafor, Fernand-Nestor Tchuenguem Fohouo

**Affiliations:** ^1^Department of Biological Sciences, Faculty of Science, University of Ngaoundéré, P.O. Box 454, Ngaoundéré, Cameroon; ^2^Department of Chemistry, Faculty of Sciences, University of Ngaoundéré, P.O. Box 454, Ngaoundéré, Cameroon; ^3^Department of Organic Chemistry, Faculty of Sciences, University of Yaoundé I, P.O. Box 812 Yaoundé, Cameroon; ^4^Laboratory of NMR and Molecular Imaging, Department of General, Organic Chemistry and Biomedical, University of Mons, B-7000 Mons, Belgium

## Abstract

Inflammatory diseases are a real public health problem worldwide. Many synthetic drugs used in the treatment of inflammatory diseases such as steroidal anti-inflammatory drugs, nonsteroidal anti-inflammatory drugs (NSAIDs) and immunosuppressive drugs have harmful side effects. However, there are natural products like propolis, which is traditionally used in the treatment of pain. The objective of this work was to evaluate the anti-inflammatory and analgesic activities of the ethyl ester of arachic acid, a compound isolated from Cameroonian propolis. The ethyl ester of arachic acid was isolated by chromatography of the ethanolic extract of propolis harvested at Tala-Mokolo (Far North Region of Cameroon) and identified by nuclear magnetic resonance (NMR) spectra and the ^1^H-^1^H correlated spectroscopy. The anti-inflammatory and analgesic properties of oral administration of arachic acid ethyl ester (12.5, 25.0, and 50.0 mg/kg bw) were evaluated using carrageenan-induced paw edema, xylene-induced ear edema, cotton pellets-induced granuloma formation, and hot plate test in rat. Arachic acid ethyl ester produced maximum inhibition at 50.0 mg/kg for carrageenan-induced paw edema (62.5%), xylene-induced ear edema (54.5%), cotton pellet-induced granuloma (47.4%), and increased mean latency for hot plate test in rats. These results show clearly that the arachic acid ethyl ester has acute and chronic anti-inflammatory properties as well as central analgesic properties. This justifies the use of propolis in the treatment of pain in traditional medicine.

## 1. Introduction

Rheumatic diseases associated with inflammatory diseases are very common worldwide and constitute a major public health problem. Inflammatory diseases are mammalian tissue pathologies caused by various agents, including infectious microorganisms, toxic chemicals, physical lesions, or tumor growth [1]. During the inflammatory process, injured tissue cells, phagocytes, lymphocytes, and mast cells secrete mediators of inflammation such as histamine, kinins, prostaglandins, complement, and lymphokines. Inflammation is a nonspecific body's response to pathogens. Acute inflammation is manifested by pain, heat, redness, swelling, and loss of function [2]. Inflammatory diseases can be acute or chronic. The body's initial response to inflammatory agents is acute inflammation characterized by increased movements of leukocytes (granulocytes) and plasma from the blood to injured tissues. During acute inflammation, several biochemical mechanisms involving the local vascular system, the immune system, and various cells occur within the injured tissue. Long-term inflammation, also known as chronic inflammation, is characterized by the simultaneous destruction and healing tissues, resulting in a gradual change in the type of cells present at the site of inflammation [3]. Many synthetic chemicals such as steroidal drugs, nonsteroidal anti-inflammatory drugs (NSAIDs), and immunosuppressive drugs available for the treatment of inflammatory diseases have some harmful side effects [4]. Propolis is a crude compound of beehive made by bees from resin harvest to several plants [5]. It is used in folk medicine as antibiotic [6], antiviral [7], antioxidant [5], and anti-inflammatory [8]. The present study was undertaken to evaluate the anti-inflammatory and analgesic activities of arachic acid ethyl ester (AAEE) isolated from propolis in rats.

## 2. Materials and Methods

### 2.1. Biological Material

#### 2.1.1. Arachic Acid Ethyl Ester Isolation and Identification

*(1) Propolis Collection*. Propolis was collected in January 2012 from Tala-Mokolo (Far North Region of Cameroon), an agroecological zone, and was identified by a Beekeeper, Damatal.

*(2) Propolis Extraction*. Dried powdered propolis (405 g) was extracted three times by maceration at room temperature with ethanol (EtOH) (3 × 6 L) for 72 hours. The supernatants were filtered and evaporated under vacuum by means of a rotary evaporator (Büch, 461) to obtain a dried brown extract (75 g).

*(3) Isolation and Identification*. The ethanol extract (75 g) was subjected to silica gel column chromatography (Ø 0.063-0.200 mm, 650 g) and eluted with the mixture n-hexane-ethyl acetate (EtOAc) and EtOAc-methanol (MeOH) in order to increase polarity (0-100%) to yield a total of 309 fractions of 300 mL each. These fractions were combined on the basis of thin-layer chromatography (TLC) analysis in fifteen major fractions (**F**_**1**_-**F**_**15**_). Fraction **F**_**1**_ was purified by silica gel column chromatography (Ø 0.063-0.200 mm, 75 g) under isocratic elution with the mixtures n-hexane-EtOAc and EtOAc-MeOH with gradient polarity (0-100%) as eluents. After filtration and crystallization, 1300 mg of the white crystal was obtained and indexed PEN_4_ and then subjected to spectral analysis. ^1^H and ^13^C nuclear magnetic resonance (NMR) spectra and ^1^H-^1^H correlated spectroscopy (COSY), distortionless enhancement by polarization transfer (DEPT), heteronuclear single quantum coherence (HSQC), and heteronuclear multiple bond correlation (HMBC) spectra were recorded from Bruker Advance DMX 400 MHz spectrometers.

**Figure 1:**
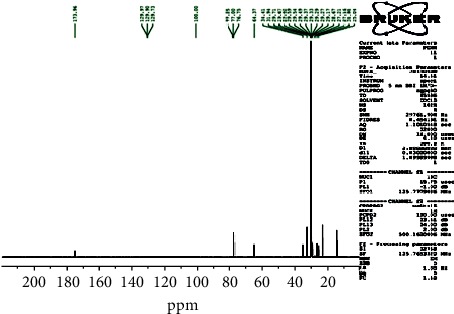
^13^C NMR spectrum (CDCl3, 125 MHz) of PEN_4_.

**Figure 2:**
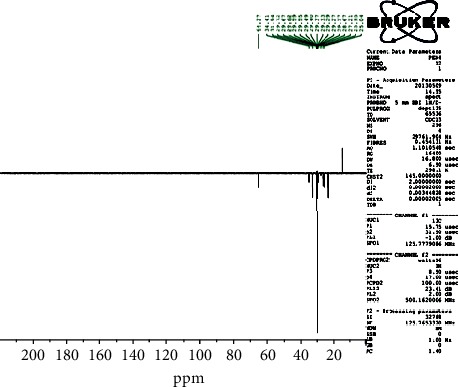
^13^C DEPT 135 spectrum (CDCl3, 125 MHz) of PEN_4_.

**Figure 3:**
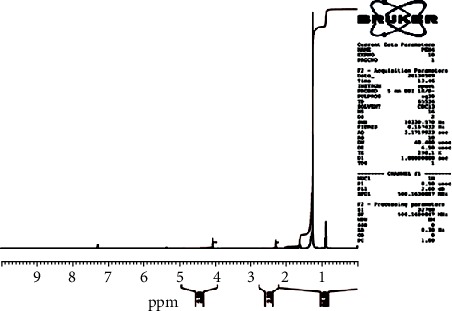
^1^H NMR spectrum (CDCl3_,_ 500 MHz) of PEN_4_.

The ^13^C-NMR spectrum (Figure 1) and ^13^C-DEPT 135 spectrum (Figure 2) indicate 22 carbons: two methyls into final position with *δ*_C_ 15.2 (C-20 and C-2′); eighteen methylenes between 25.04 ppm and 34.41 ppm; one oxygenated methyl with *δ*_C_ 64.37 (C-1′); and one quaternary carbon with *δ*_C_ 173.96 (C-1), ascribable to the carbon of the function ester. Moreover, the ^1^H-NMR spectrum (Figure 3) showed a triplet with *δ*_H_ 0.88 ascribable to the protons of the two groupings methyls, proton of methyl in *α* (*δ*_H_ 2.26/H-2) and proton of methyl in *β* (*δ*_H_ 1.58/H-3) of the function ester, and protons of O-methyl with *δ*_H_ 4.1 (H-1′), and an aliphatic methyl long chain have *δ*_H_ 1.35 (CH_2_)_16_.

**Figure 4:**
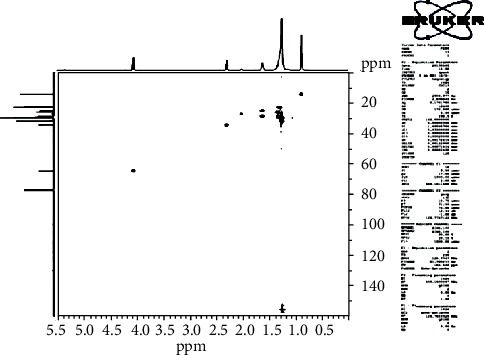
HSQC spectrum (CDCl3, 125 MHz) of PEN_4_.

The HSQC experiment showed the protons with *δ*_H_ 4.1 (H-1′) that were related to carbon with *δ*_C_ 64.37 (C-1′), the protons in *α* (*δ*_H_ 2.26/H-2) of the function ester related to carbon with *δ*_C_ 34.41 (C-2), the protons in *β* (*δ*_H_ 1.58/H-3) of the function ester related to carbon with *δ*_C_ 25.95 (C-3), and the protons with *δ*_H_ 0.88 attached to carbons with *δ*_C_ 15.2 (Figure 4).

**Figure 5:**
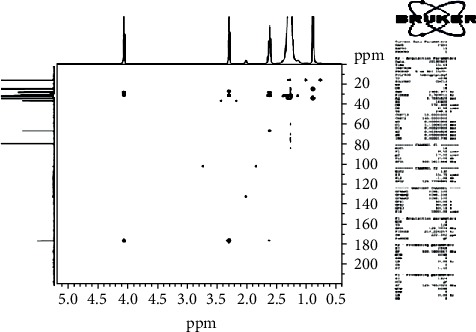
HMBC spectrum (CDCl3, 125 MHz) of PEN_4_.

The HMBC spectrum (Figure 5) showed a correlation between protons of the O-methyl (H-1′) and carbons with *δ*_C_ 173.96 (C-1), *δ*_C_ 25.95 (C-3), protons in *α* (*δ*_H_ 2.26/H-2) and *β* (*δ*_H_ 1.58/H-3) of the function ester, and protons of two methyls and carbons with *δ*_C_ 25.04 (C-19) and *δ*_C_ 34.41 (C-2), respectively.

**Figure 6:**
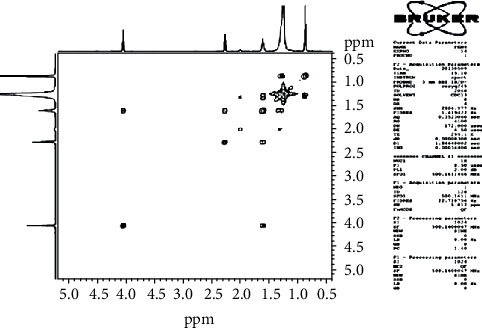
COSY spectrum of PEN_4_.

The COSY spectrum (Figure 6) showed correlations between protons of the O-methyl (*δ*_H_ 4.1/H-1′) and protons in *β* (*δ*_H_ 1.58/H-3) of the function ester, protons in *β* (*δ*_H_ 1.58/H-3) and *α* (*δ*_H_ 2.26H-2) of the function ester, protons with *δ*_H_ 0.88/H-20, and protons with *δ*_H_ 1.35/H-19.

**Figure 7:**
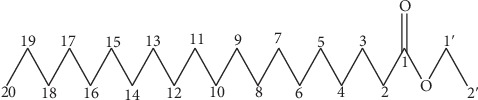
Ethyl arachidate or arachic acid ethyl ester (AAEE) structure.

A combination of NMR, DETP, COSY, HSQC, and HMBC spectral data, compared with those of the literature [9, 10], makes it possible to give to the made-up PEN_4_, the structure which is that of the ethyl arachidate or arachic/arachidic acid ethyl ester (AAEE) with a formula C_22_H_44_O_2_ (Figure 7). Within the limit of our knowledge, the compound is insulated for the first time from the propolis.

Prior to the oral administration, AAEE was dissolved in refined palm oil so that each animal received less than 10 mL/kg body weight solution.

#### 2.1.2. Animals

Wistar albino rats (150–180 g), obtained from the breeding house of the University of Ngaoundéré, were allowed water and food ad libitum. The rats were kept in standard polypropylene cages (five/cage). All animals were acclimatized for one week under laboratory environmental conditions (temperature and dark/light cycle) before the start of the study. *In vivo* experimental treatments of animals have been carried out in accordance with the European Union guidelines on Animal Care (CEE Council 86/609) [11] that was adopted in Cameroon by the Institutional Committee of the Ministry of Scientific Research and Innovation.

### 2.2. Anti-Inflammatory Test

#### 2.2.1. Carrageenan-Induced Paw Edema

The carrageenan-induced paw edema model rat used has been previously described by the Winter et al. method [12]. Briefly, 25 rats were randomly divided into five groups of five rats each and treated as follows:

1. Group 1 (negative control group: NC) received palm oil as vehicle (10 mL/kg)
2. Groups 2 to 4 (test groups), respectively, received arachic acid ethyl ester 12.5 mg/kg bw (AAEE12.5), arachic acid ethyl ester 25 mg/kg bw (AAEE25), and arachic acid ethyl ester 50 mg/kg bw (AAEE50)
3. Group 5 (positive control group: Dexa5) received dexamethasone 5 mg/kg bw as reference drugs

One hour after this treatment, inflammation was induced by subplantar injection of 0.1 mL of 1% suspension of carrageenan (Sigma Chemical Co., St Louis, USA) in normal saline (9%), in the right hind paw of each rat. Edema formations were measured immediately prior to the injection of carrageenan and thereafter at intervals of 1 for 6 h. The inhibition of edema was calculated according to the following formula [3]:

(1) $$\begin{array}{c} \text{Percentage inhibition}=\frac{\left(C_{t}-C_{0}\right)\text{Untreated}-\left(C_{t}-C_{0}\right)\text{Treated}}{\left(C_{t}-C_{0}\right)\text{Untreated}}\times 100, \end{array}$$

where *C*_*t*_ means the paw circumference for each group at time *t* and *C*_0_ means the paw circumference for each group before carrageenan injection.

#### 2.2.2. Xylene-Induced Ear Edema

Five groups of five normal rats each received *per os* one hour before xylene application, arachic acid ethyl ester (AAEE) at dose 12.5 mg/kg (AAEE12), 25 mg/kg (AAEE25), and 50 mg/kg (AAEE50) body weight, dexamethasone 2.5 mg/kg (Dexa5) as a positive control or palm oil (10 mL/kg) as a negative control.

Ear edema was induced by applying 0.03 mL of xylene to the posterior and anterior surfaces of the right ear of each rat. The left ear did not receive xylene and was considered as control. The animals were then anesthetized with diethyl ether, and the right and left ears were perforated (9 mm in diameter) using a borer. Each ear punch was weighed and the differences between the weight of the right ear and the left ear punches of each rat were calculated [4].

#### 2.2.3. Cotton Pellet-Induced Granuloma Formation in Rat

Five groups of five normal rats each were anesthetized with diethyl ether. Sterilized cotton pellet (20 mg) was then surgically implanted subcutaneously in both shaved axilla regions of rats using small incisions. The control group (Group 1) received orally refined palm oil (10 mL/kg), the test groups (Group 2, Group 3, and Group 4) received orally arachic acid ethyl ester 12.5, 25, and 50 mg/kg bw, respectively, and the fifth group received oral morphine at 5 mg/kg body weight, and this was done once daily for a period 7 consecutive days. At the eighth day, cotton pellets were dissected out under ether anesthesia, cleaned of extraneous tissue, weighed, and dried at 60°C to a constant weight. The mean weight for different groups was determined. The measurement of granuloma formation was appreciated by increasing the dry weight of pellets [13].

### 2.3. Analgesic Test

Five groups of five rats each were used to study the analgesic activity of arachic acid ethyl ester by a hot plate test (pain model). The control group (Group 1) received refined palm oil (10 mL/kg), the test groups (Group 2, Group 3, and Group 4) received arachic acid ethyl ester 12.5, 25, and 50 mg/kg bw, respectively, and group five received morphine at 2.5 mg/kg bw. The rats were placed on Eddy's hot plate and maintained at a temperature of 55 ± 0.5°C. Hot plate latencies were measured at 0, 0.5, 1, 2, 3, 4, 5, and 6 hours after treatment [14]. Animals were delayed not more than 30 seconds every time on the hot plate [15]. The analgesic activity was calculated according to formula

(2) $$\begin{array}{c} \text{PA }\left(\%\right)=\frac{\left(\text{MTR treated}\right)-\left(\text{MTR untreated}\right)}{\left(\text{MTR treated}\right)}\times 100, \end{array}$$

where MTR means the time of reaction and PA the percentage of analgesic activity.

### 2.4. Statistical Analysis

Data were expressed as mean ± standard error of mean ($\bar{\text{X}}\pm \text{SEM}$). The data were analyzed by one-way ANOVA followed by Dunnett's *t*-test using the computerized GraphPad InStat 3.05 versions (GraphPad Software, USA). Differences were considered significant when *p* < 0.05.

## 3. Results

### 3.1. Effect of Arachic Acid Ethyl Ester (AAEE) on Carrageenan-Induced Paw Edema in Rats

**Figure 8:**
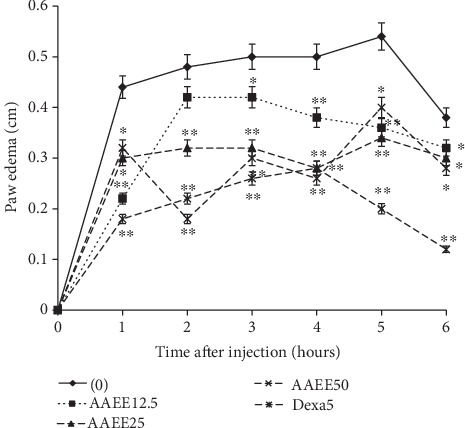
Effect of arachic acid ethyl ester (AAEE) on the carrageenan-induced paw edema in rat. Values are expressed as mean ± SEM (*n* = 5). Significant difference: ^∗^*p* < 0.05 and ^∗∗^*p* < 0.01 compared with control at the same time point. Dexa5: dexamethasone.

The injection of carrageenan resulted in an increase of paw edema between the first hour (0.44 cm) and the fifth hour (0.54 cm) but falls at the sixth hour (0.38 cm) in the control group. These values decreased in the treated group over time. They decreased significantly (*p* < 0.01) from the first hour to the sixth hour in all treated groups (Figure 8). Maximum inhibitions were 62.50% in the group treated with AAEE 50 mg/kg bw at the second hour and 68.22% in the group treated with dexamethasone 5 mg/kg bw.

### 3.2. Effect of Arachic Acid Ethyl Ester (AAEE) on Xylene-Induced Ear Edema in Rat

In the untreated group (control) rats, ear edema induced by xylene was 6.6 ± 0.3 mg. In rats treated with the AAEE solution at doses of 12.5 mg/kg, 25 mg/kg, and 50 mg/kg bw or treated with dexamethasone 5 mg/kg bw, xylene-induced ear edema significantly reduced (*p* < 0.01) and were, respectively, 4.0 ± 0.2 mg, 3.2 ± 0.1 mg, 3.0 ± 0.1 mg, and 2.8 ± 0.1 mg (Table 1). Maximum inhibitions were 54.55% in the group treated with AAEE 50 mg/kg bw at the second hour and 57.58% in the group treated with dexamethasone 5 mg/kg bw.

### 3.3. Effect of Arachic Acid Ethyl Ester (AAEE) on Cotton Pellet-Induced Granuloma Formation in Rats

In the untreated group (control) rats, granuloma formation in rats induced by cotton pellet was 57.2 ± 3.1 mg. In rats treated with the AAEE solution at doses of 12.5 mg/kg, 25 mg/kg, and 50 mg/kg bw or treated with dexamethasone 5 mg/kg bw, the cotton pellet-induced granuloma formulation significantly reduced (*p* < 0.01) and were 45.3 ± 2.1 mg, 36.0 ± 2.2 mg, 30.2 ± 1.0 mg, and 27 ± 1 mg, respectively (Table 2). Maximum inhibitions were 47.4% in the group treated with AAEE 50 mg/kg bw at the second hour and 52.6% in the group treated with dexamethasone 5 mg/kg bw.

### 3.4. Analgesic Effect of Arachic Acid Ethyl Ester (AAEE)

In the hot plate method, both AAEE and morphine were found to exhibit a dose-dependent increase significantly (*p* < 0.01) in latency time when compared with the control group. The latency period at different times has increased significantly compared to the initial values in the same treated group. The maximal effect of the drug was observed at a dose of 50 mg/kg, which gave a maximal latency time of 26.0 ± 6.8 s, 2 h before administering drugs with a maximal analgesic percentage of 80.62% similar to morphine (2.5 mg/kg) with 25.4 ± 6.8 s of maximal reaction and an analgesic percentage of 80% (Table 3).

## 4. Discussion

There are two main types of anti-inflammatory drugs: steroidal anti-inflammatory drugs (SAIDs) and nonsteroidal anti-inflammatory drugs (NSAIDs) [16]. Nonsteroidal anti-inflammatory drugs (NSAIDs) work by inhibiting the activity of the enzyme cyclooxygenase (COX-1 and COX-2) and suppressing the formation of prostaglandins responsible for pain and edema [17, 18]. Arachic acid ethyl ester was effected in a rat model of acute and chronic inflammation as well as analgesic activities.

Carrageenan induces inflammation by the prostaglandins synthesis responsible for pain and edema. Nonsteroidal anti-inflammatory agents primarily inhibit the cyclooxygenase involved in the synthesis of these prostaglandins [19]. Carrageenan-induced edema has been commonly used as an animal model for inflammation and is believed to be biphasic [20]. During the early phase of inflammation, damaged and surrounding tissues synthesize mainly mediators such as histamine, serotonin, and a large amount of prostaglandins. The release of these prostaglandins supports the terminal phase of inflammation mediated by bradykinin, leukotrienes, polymorphic nuclear cells, and prostaglandins produced by tissue macrophages [21]. In this study, arachic acid ethyl ester significantly elicited inhibitory effect on edema formation at all assessment time, similar to that exhibited by the group treated with dexamethasone. These results indicate that this compound can act either by inhibiting the synthesis and release of inflammatory mediators such as prostaglandins, histamine, serotonin, and bradykinin or by inhibiting the activity of cyclooxygenase.

Xylene induces inflammation by increasing the activity of phospholipase A_2_ which can be inhibited by topical anti-inflammatory steroids or nonsteroidal antiphlogistic agents [22]. This acute model was mediated by histamine, serotonin, and bradykinin. In the present study, the increase in ear weight was dose-dependently inhibited by the arachic acid ethyl ester treatment. This compound would oppose the secretion or action of inflammatory mediators, thereby confirming the anti-inflammatory effect observed during the first phase of carrageenin-induced paw edema in rat [4].

The cotton pellet granuloma model was used to evaluate the activity of ethyl arachidate in chronic inflammation. A cotton pellet implanted subcutaneously in the rat induces inflammation in three phases: the first phase (transudative phase), which lasts about 3 hours, the second phase (exudative phase) which takes place between 3 and 72 hours after the implantation of the pellet, and the third phase (proliferative phase) characterized by the increase in the dry weight of the granuloma and which occurs between 3 days and 6 days after implantation [19], due to proliferation of inflammatory cells like macrophages, neutrophils, and fibroblasts which accumulate at the site implant [23]. In this model, arachic acid ethyl ester effectively inhibited the development of granulomatous tissues compared to control group. This isolated compound may act by inhibiting neutrophils and macrophages migration or by inhibiting fibroblast and the collagen synthesis, which are natural proliferative events of granuloma formation [24].

Rodents' feet are sensitive to heat at temperatures that do not damage the skin. The responses of these rodents to this heat are jumping, removing legs, and/or licking paws [18]. A number of complex processes, such as opiate, noradrenergic, dopaminergic, and serotonergic systems, control pain [18, 19]. Arachic acid ethyl ester increase considerably latency time of rats on hot plate according all assessment time similar to morphine (2.5 mg/kg), a reference analgesic opioid which elevated the heat threshold without major inhibitory effect on psychomotor activity [25]. According to the results of this study, we can conclude that this isolated compound possesses centrally acting analgesic by the inhibition of pain sensation.

Arachidonic acid is one of the major polyenoic fatty acids in mammals. It is the precursor of an important group of biologically active compounds such as prostaglandins, prostacyclins, thromboxanes, and leukotrienes which are responsible for the inflammatory process and pain. These were mediated by cyclooxygenase or lipoxygenase [17]. Arachidic acid is a saturated fatty acid which has the same carbon number C_20_ as the unsaturated arachidonic acid. Ethyl arachidate would acted by competitive inhibition on the cyclooxygenase and lipoxygenase such as NDAIDs.

## 5. Conclusion

Arachic acid ethyl ester would act as an inhibitor of the action of cyclooxygenase and/or lipoxygenase. These results show clearly that the arachic acid ethyl ester, isolated in the propolis, has acute and chronic anti-inflammatory properties as well as central analgesic properties. This justifies the use of propolis in the traditional treatment of pain.

## Figures and Tables

**Table 1:** Effect of arachic acid ethyl ester (AAEE) on xylene-induced ear edema in rat.

Group	Treatment	Weight of ear edema (mg)	% inhibition
Control	PO (10 mL/kg)	6.6 ± 0.3	
Dexamethasone	5 mg/kg	2.8±0.1^∗∗^	57.6.6
AAEE	12.5 mg/kg	4.0 ± 0.2^∗^	39.4
	25 mg/kg	3.2±0.1^∗∗^	51.5
	50 mg/kg	3.0±0.1^∗∗^	54.5

Values are expressed as mean ± SEM (*n* = 5). Significant difference: ^∗^*p* < 0.05 and ^∗∗^*p* < 0.01 compared with control. PO: palm oil.

**Table 2:** Effect of arachic acid ethyl ester (AAEE) on cotton pellet-induced granuloma in rats.

Group	Treatment	Granuloma weight (mg)	% inhibition
Control	PO (10 mL/kg)	57.2 ± 3.1	
Dexamethasone	5 mg/kg	27.1±1.2^∗∗^	52.6
AAEE	12.5 mg/kg	45.3 ± 2.1^∗^	21.1
	25 mg/kg	36.0±2.2^∗∗^	36.8
	50 mg/kg	30.2±1.0^∗∗^	47.4

Values are expressed as mean ± SEM (*n* = 5). Significant difference: ^∗^*p* < 0.05 and ^∗∗^*p* < 0.01 compared with control. PO: palm oil.

**Table 3:** Analgesic activity of arachic acid ethyl ester (AAEE) induced by the hot plate method in rats.

Treatment		Time (hour)							
		0	0.5	1	2	3	4	5	6
Palm oil (mL/kg)	10	6.8 ± 1.9	5.0 ± 1.0	6.2 ± 2.7	5.2 ± 1.1	5.4 ± 1.8	5.0 ± 1.7	5.0 ± 0.7	5.0 ± 0.7
Morphine (mg/kg)	2.5	5.8 ± 3.5	7.6 ± 4.0 (34)	20.0 ± 5.9^∗^ (69)	22.0 ± 7.2^∗^ (76)	25.4±6.8^∗∗^ (79)	25.2±5.7^∗∗^ (80)	24.8 ± 7, 8^∗∗^ (80)	20.8±7.3^∗∗^ (76)
AAEE (mg/kg)	12.5	6.6 ± 2.1	10.6 ± 3.4^∗^ (53)	12.2 ± 3.0 (49)	11.2 ± 5.8 (54)	13.6 ± 6.1 (60)	13.2 ± 6.9 (62)	7.4 ± 2.3 (32)	7.2 ± 1.9 (31)
	25	6.8 ± 1.1	13.2 ± 3.7^∗^ (62)	13.6 ± 3.0 (54)	14.4 ± 3.7 (64)	12.4 ± 3.2 (56)	10.4 ± 2.9 (52)	8.0 ± 3.2 (37)	8.4 ± 1.9 (40)
	50	6.4 ± 1.7	7.0 ± 1.2 (29)	20.2 ± 9.2^∗^ (69)	26.0±6.8^∗∗^ (80)	22.4 ± 7.0^∗^ (76)	19.8 ± 6.8^∗^ (75)	24.8±7.6^∗∗^ (80)	25.8±7.8^∗∗^ (81)

Each value is the mean of latency time (s) on the hot plate ± SEM (*n* = 5). Significant difference: ^∗^*p* < 0.05 and ^∗∗^*p* < 0.01 compared with control at the same time point. (): % of analgesic activity.

## Data Availability

The data used to support the findings of this study are available from the corresponding author upon request.
